# Monochorionic Triplet Gestation after Single Blastocyst Transfer Using Donor Oocytes: Case Report and Review

**DOI:** 10.1155/2020/4340617

**Published:** 2020-07-28

**Authors:** G. Ferri, M. Musto, G. Colombo, V. M. Savasi

**Affiliations:** Unit of Obstetrics and Gynecology, Department of Biomedical and Clinical Sciences, ASST Fatebenefratelli Sacco, Hospital “L. Sacco”, University of Milan, Via GB Grassi 74, 20157 Milan, Italy

## Abstract

We report a case of a 43-year-old patient with a monochorionic triamniotic triplet pregnancy after IVF with donor oocytes. After failed IVF attempts, the patient chose to undergo treatment with donor oocytes. Her 22-year-old oocyte donor underwent standard controlled ovarian hyperstimulation. The retrieved oocytes were fertilized in vitro, and one embryo was transferred at the blastocyst stage. At 6 weeks and 5 days of gestation, an ultrasound revealed monochorionic triamniotic (MCTA) triplets. The risk of monozygotic twinning in women undergoing in vitro fertilization (IVF) is reported to be higher than that in natural conception, although the causes of the phenomenon have not yet been clarified. Efforts still must be made in order to prevent monozygotic multiple pregnancies, associated with much greater chances of obstetric and perinatal morbidity and mortality.

## 1. Introduction

The major goal of in vitro fertilization (IVF), today, is to obtain the birth of a healthy singleton. In order to achieve this target, a reduction in the number of embryos transferred during assisted reproduction has been performed worldwide in the last few years. Despite this, the prevalence of zygotic splitting and monozygotic multiple pregnancies persists [1]. Since the first report of an IVF monozygotic twinning (MZT) pregnancy in 1984 by Yovich et al., approximately 1.7% of all clinical IVF pregnancies have been diagnosed as MZT. The risk of monozygotic twinning in women undergoing in vitro fertilization (IVF) is reported to be higher than that in natural conception (1.7% vs. 0.4%, respectively). The incidence of monozygotic triplet pregnancies has been reported to occur in 0.048% of IVF pregnancies, 100 times more than that in natural conception, which is stated to be 1 : 100000.

There are no data on the difference of incidence of monozygotic multiple pregnancy in donor oocyte and homologous pregnancies.

Despite the increased incidence after ART, monozygotic triplets are still rare, and to the best of our knowledge, there have been 20 reports of MCTA triplets published in the literature since 1994 (Table 1), and only 6 resulted from the transfer of a single embryo.

In this publication, we report a case of MCTA triplets following IVF with donor oocytes which we believe to be the first reported with assisted reproductive technique (ART) in Italy.

## 2. Case Report

A 43-year-old Caucasian presented with her partner for IVF with donor oocytes. Her reproductive history included two missed miscarriage at 8 weeks of pregnancy in the past. In her history, there was no significant pathology. She was a nonsmoker, took no regular medications, and used alcohol only rarely. Her only regular medication was a prenatal acid folic. Physical exam revealed no abnormality; BMI was 20 kg/m^2^. The study of female fertility showed a poor ovarian reserve. A normal uterine cavity had been investigated with hysteroscopy. None of the partners had STDs. The study of male fertility showed mild asthenoteratozoospermia, with a normal DNA fragmentation test. The male partner was also in good health, and there was no significant pathology. The urethral swabs were negative. The oocyte donor was a 22-year-old.

The egg donation was performed in a private clinic in Spain. Controlled ovarian hyperstimulation of the egg donor was performed by using a combined FSH+hMG protocol. Transvaginal ultrasound-guided aspiration of oocytes yielded 15 oocytes. 14 oocytes were fertilized with conventional IVF, and after 5 days of in vitro culture, 6 blastocysts were obtained. One top quality blastocyst was transferred and 5 were cryopreserved. A negative beta-hCG was obtained 14 days after embryo transfer.

After two months, a frozen embryo transfer was programmed. The endometrium was prepared with 2 mg of progynova 3 times a day from the 2nd day of the menstrual cycle. The embryo transfer was programmed when the endometrial thickness was greater than 8 and the blood progesterone was 15 ng/ml.

A positive beta-hCG was obtained 14 days after embryo transfer. She received luteal support with vaginal progesterone (Progeffik 200 mg, 1 vaginal tablet in the morning and 2 at night).

**Figure 1:**
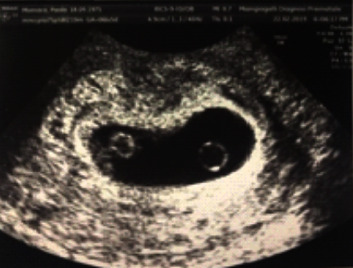
Two-dimensional scan showing three yolk sacs and a single placenta.

At 6 weeks and 5 days of gestation, an ultrasound revealed monochorionic triamniotic (MCTA) triplets with three yolk sacs and three fetal poles. The cardiac activity was visible only for two fetal poles (Figure 1).

A transvaginal ultrasound at 8 weeks and 4 days of gestation revealed fetal activity just for a fetus.

At 12 weeks and 2 days of gestation, a noninvasive screening test was programmed. The nuchal translucency was 1.4 mm, the nasal bone was present, and velocimetry of the venous duct was normal. The maternal biochemical parameter was tested (PAPPA-A mom 3369 and free beta-hCG mom 1856). The uterine artery mean PI was >95 percentile.

Therapy with 150 mg pro die ASA was prescribed to reduce the risk of preeclampsia [10]. The test (age donor, nuchal translucency, and biochemical value of PAPPA-A and free beta-hCG) showed a lower risk at the threshold value of 1 : 250 for all the chromosomopathies investigated (trisomies 21, 13, and 18) [11].

A transabdominal ultrasound at 16 weeks of gestation revealed the absence of cardiac activity and biometry corresponding to 13 weeks of gestation. The revision of the uterine cavity was programmed, and the result of the histological examination showed necrotic-hemorrhagic decidua and chorionic villi in diffuse villous involution. No pathological or malformative changes were found against the embryo parts.

## 3. Discussion

To our knowledge, this is the first report of monozygotic triplet pregnancy reported in Italy.

The mechanism of zygotic splitting has been debated, but an explanation has not been found yet despite the fact that multiple studies have been made. Investigations of potential risk factors, such as the age of the oocyte, extended culture, and assisted hatching (AH), have provided no clear explanation of the aetiology. The first and most recognized theory considers monozygosity caused by manipulation of the zona pellucida such as spermatic microinjection (ICSI) and assisted hatching (AH). Theoretically, twinning could result from herniation of blastomeres through the hole of the zona pellucida and embryo splitting during blastocyst expansion (Alikani et al., 1994; da Costa et al., 2001; Skiadas et al., 2008; Saravelos et al., 2016). However, recent studies did not find an association between MZT and ICSI/AH (Franasiak et al., Knopman et al., Matezel et al., Milki et al., Papanikolaou et al.). Further studies have shown embryo manipulation using the procedures of blastocyst transfer, AF, and frozen-warmed ET, and also genetic factors, ovarian stimulation, embryo quality, and younger maternal age are potential risk factors affecting embryo division. The most consistent risk factor is considered to be extended blastocyst culture which more often leads to zygote splitting compared to cleavage stage embryo (Steinman and Valderrama, 2001; Menezo and Sakkas, 2002; [12], Sharara and Abdo, 2010; Kawachiya et al., 2011; Sotiroska et al., 2015). Indeed, out of the 20 MZT we reported in our table, 12 were blastocyst transfers (60%). The majority of recent studies have reported a significant influence of maternal age on the MZT rate showing an increase in the MZT rate in cases of young maternal age (Franasiak et al., Kawachiya et al., Knopman et al., Sotiroska et al.)

A retrospective study was performed by Hanyan et al. in order to analyze the risk factors associated with IVF-conceived monozygotic twinning. Out of 3463 IVF pregnancies, ninety-three women (2.69%) with MZT were observed. The results showed that increased rates of MZT in fresh and frozen cycles are significantly associated with extended culture, confirming previous reports stating that MZT is associated with prolonged embryo culture. Simultaneously, no significant difference in the incidence of MZT between the fresh and frozen cycles and no effect of AH and ICSI on the risk of MZT were demonstrated. In agreement with prior reports, there was an increased rate of MZT with blastocyst transfer, which is generally thought to be correlated with younger oocytes and better embryo quality.

A recent meta-analysis (Hviid et al., 2018) including systemic reviews, meta-analysis, and original studies tried to identify the risk factors associated with MZT after IVF and ICSI. Data support a higher rate of MZT after blastocyst transfer compared with cleavage stage transfer and the highest risk of MZT with younger maternal age or with the use of younger oocytes because of their better quality compared with older oocytes.

## 4. Conclusion

Despite the increased incidence after ART, monozygotic triplets are still rare and consequently it is difficult to identify the associated ART procedures that lead to their occurrence. Data from multiple studies support a higher rate of MZT after blastocyst transfer compared with cleavage stage transfer and the highest risk of MZT with younger maternal age or with the use of younger oocytes because of their better quality compared with older oocytes. Advances in cell culture media have led to a shift in in vitro fertilization (IVF) practice from cleavage stage embryo transfer to blastocyst stage transfer. The rationale for blastocyst transfer is to improve both uterine and embryonic synchronicity and enable self-selection of viable embryos, thus resulting in better live birth rates. Considering the higher risk of MZT associated with younger oocytes and blastocyst transfer, could we think of transferring cleavage stage embryo in younger women as a possible solution?

Efforts still must be made in order to prevent monozygotic multiple pregnancies, associated with much greater chances of obstetric and perinatal morbidity and mortality.

## Figures and Tables

**Table 1:** Published case reports of MCTA triplets following ART.

Author and year of publication	Age	No. of embryos transferred	Transfer day	IVF/ICSI	Outcome
Salat-Baroux et al. 1994 [2]	26	4	3	IVF	Week 6: fetal reduction of triplets Week 13: spontaneous abortion of twins
Belaisch-Allart et al. 1995 [3]	37	3	3	IVF	Week 35: CS-BW: 2000, 2000, 2380 g
Yakin et al. 2001	34	3	5	ICSI	Week 10: selective reduction of one triplet
Ghulmiyyah et al. 2003 [4]	21	3	3	ICSI	Week 30: CS due to preeclampsia BW: 1475, 1021, 1021 g
Zikopoulos et al. 2004	39	2	5	ICSI	Week 11: embryo reduction of triplets Week 20: uneventful twin gestation
Risquez et al. 2004	40	1	3	ICSI	Week 16: at the time of writing, gestation is uneventful
Unger et al. 2004	38	2	5	ICSI	Week 11: selective fetal reduction of one triplet Week 22: twin gestation continuing
Jain et al. 2004 [5]	23	2	5	IVF	Week 8: cardiac motion ceased in one of the triplets Week 32: CS identical female twins-no problems
Ulug et al. 2004	30	2	3	ICSI	Week 34: healthy female babies BW: 1960, 1870, and 1530
Henne et al. 2005 [6]	23 ed	2 blastocysts Egg donation	5	IVF	Termination due to high risk
Yanaihara et al. 2007	39	1 blastocyst	5	IVF	Week 9: terminated pregnancy
Faraj et al. 2008 [7]	27 ed	1 blastocysts Egg donation	5	IVF	Week 32: CS-BW: 1695, 1560, 1500 g
Lee et al. 2008	28	1 blastocyst	5	ICSI	Week 33: CS-BW: 1780, 1780, 1780 g No respiratory problems
Pantos et al. 2009	34	3 embryos	4	ICSI	MZTP+MCMA Twin selective reduction of the MZTP. Fetal reduction to singleton pregnancy Week 38: CS-BW: one healthy male
Dessolle et al. 2010 [8]	27	1 blastocyst	5	ICSI	Week 16: reduction to twin pregnancy Week 19: severe TTTS Week 34: CS-BW: 1570, 1710 g
Dessolle et al. 2010 [8]	30	1 blastocyst	5	IVF	Week 15: reduction to twin pregnancy Week 34: CS-BW: 1320, 1970 g
Tal et al. 2012 [9]	29	3 blastocysts	3	IVF	Fetal reduction of triplets and CS of twins
Radwan et al. 2014	32	2 blastocysts	5	IVF	Week 33: CS-BW: 2060, 1860, 2000 g
Gurunath et al. 2015 [1]	29	2 blastocysts	5	IVF	Week 17: pPROM and cord prolapse
Xiang-Lin Li 2018	35	2 morulas	4	IVF	Week 33: CS-BW: 1235, 1880, 1855 g

CS-BW: cesarean section-birth weight; TTTS: twin-to-twin transfusion syndrome; MZTP: monozygotic triplet pregnancy; MCMA: monochorionic monoamniotic; ed: egg donor.
